# Outcomes of Modified Mayo Stage IIIa and IIIb Cardiac Light-Chain Amyloidosis: Real-World Experience in Clinical Characteristics and Treatment—67 Patients Multicenter Analysis

**DOI:** 10.3390/cancers16081592

**Published:** 2024-04-21

**Authors:** Grzegorz Charliński, Maximilian Steinhardt, Leo Rasche, Veronica Gonzalez-Calle, Camila Peña, Harsh Parmar, Katarzyna Wiśniewska-Piąty, Julio Dávila Valls, Magdalena Olszewska-Szopa, Lidia Usnarska-Zubkiewicz, Alessandro Gozzetti, Sara Ciofini, Massimo Gentile, Elena Zamagni, Michał Kurlapski, Wojciech Legieć, David H. Vesole, Artur Jurczyszyn

**Affiliations:** 1 Department of Nephrology, Hypertension and Internal Medicine, University of Warmia and Mazury in Olsztyn, 10-719 Olsztyn, Poland; 2 Department of Hematology and Bone Marrow Transplantation, Nicolaus Copernicus Hospital, 87-100 Torun, Poland; 3 Department of Internal Medicine II, University Hospital of Wurzburg, 97080 Wurzburg, Germany; steinhardt_m@ukw.de (M.S.); rasche_l@ukw.de (L.R.); 4 Interdisciplinary Amyloidosis Center of Northern Bavaria, University Hospital Wurzburg, 97080 Wurzburg, Germany; 5 a Servicio de Hematología, Hospital Universitario de Salamanca, Instituto de Investigación Biomédica de Salamanca (IBSAL), Centro de Investigación del Cáncer de Salamanca, 37007 Salamanca, Spain; 6 Department of Hematology, Hospital del Salvador, Santiago 7500922, Chile; camipena@gmail.com; 7 Division of Multiple Myeloma, John Theurer Cancer Center at Hackensack, Meridian School of Medicine, Hackensack, NJ 07701, USA; harsh.parmar@hmhn.org; 8 Department of Hematology and Bone Marrow Transplantation, Silesian Medical University, 40-055 Katowice, Poland; k.wisniewska.piaty@gmail.com; 9 Servicio de Hematologia, Hospital Nuestra Señora de Sonsoles, 05004 Ávila, Spain; juldaval@hotmail.com; 10 Department of Hematology, Blood Neoplasms and Bone Marrow Transplantation, Wroclaw Medical University, 51-141 Wroclaw, Poland; molszopa@gmail.com (M.O.-S.); lidia.zubkiewicz@gmail.com (L.U.-Z.); 11 Hematology, Department of Medical Science, Surgery and Neuroscience, University of Siena, 53100 Siena, Italy; alegozzetti@icloud.com (A.G.); saraciofini@hotmail.it (S.C.); 12 Hematology Unit, Department of Onco-Hematology, A.O. of Cosenza, 87100 Cosenza, Italy; 13 Department of Pharmacy, Health and Nutritional Science, University of Calabria, 87036 Rende, Italy; massimogentile@virgilio.it; 14 Dipartimento di Medicina Specialistica, Diagnostica e Sperimentale, IRCCS Azienda Ospedaliero-Universitaria di Bologna, Istituto di Ematologia ‘Seràgnoli’, Università di Bologna, 40126 Bologna, Italy; e.zamagni@unibo.it; 15 Department of Hematology and Transplantology, Medical University of Gdańsk, 81-519 Gdańsk, Poland; kurlapski.michal@gmail.com; 16 Department of Hematology and Bone Marrow Transplantation, St. John of Dukla Oncology Center of Lublin Land, 20-090 Lublin, Poland; legiec.wojciech@gmail.com; 17 Division of Hematology/Oncology, Medstar Georgetown University Hospital, Washington, DC 20007, USA; david.vesole@hmhn.org; 18 Plasma Cell Dyscrasias Center, Department of Hematology, Faculty of Medicine, Jagiellonian University College of Medicine, 31-066 Kraków, Poland; mmjurczy@cyf-kr.edu.pl

**Keywords:** cardiac light-chain amyloidosis, clinical characteristics, prognostic factors, stage III, the European 2012 modification of Mayo 2004 classification, treatment

## Abstract

**Simple Summary:**

Light-chain amyloidosis (AL) is a rare multisystem disorder. One of the most common organs involved in AL is the heart. We conducted a multi-center, retrospective analysis of 67 patients with the European 2012 modification of Mayo 2004 stage III cardiac AL. The prognosis of patients with advanced cardiac amyloidosis is poor. The median OS for the entire group was 35 months (95% CI: 7–67). The most important prognostic factors with the most significant impact on OS improvement in patients with modified Mayo stage III cardiac AL identified by multivariate Cox analysis are ECOG PS ≤ 1, NYHA FC ≤ 2, and achieving hematological response ≥ VGPR and cardiac response ≥ PR after first-line treatment.

**Abstract:**

Light-chain amyloidosis (AL) is a rare multisystem disorder characterized by the deposition of misfolded amyloid fibrils derived from monoclonal immunoglobulin light chains in various organs. One of the most common organs involved in AL is the heart, with 50–70% of patients clinically symptomatic at diagnosis. We conducted a multi-center, retrospective analysis of 67 patients diagnosed between July 2012 and August 2022 with the European 2012 modification of Mayo 2004 stage III cardiac AL. The most important factors identified in the univariate Cox analysis contributing to a longer OS included Eastern Cooperative Oncology Group performance status (ECOG PS) ≤ 1, New York Heart Association functional classification (NYHA FC) ≤ 2, the use of autologous stem cell transplantation (ASCT) after induction treatment, achieving a hematological response (≥very good partial response) and cardiac (≥partial response) response after first-line treatment. The most important prognostic factors with the most significant impact on OS improvement in patients with modified Mayo stage III cardiac AL identified by multivariate Cox analysis are ECOG PS ≤ 1, NYHA FC ≤ 2, and achieving hematological response ≥ VGPR and cardiac response ≥ PR after first-line treatment.

## 1. Introduction

Light-chain amyloidosis (AL) is a rare, multisystem disease caused by the deposition of misfolded amyloid fibrils in internal organs derived from monoclonal immunoglobulin light chains [1,2]. The median age at diagnosis is 63 years, and 55% of patients are male. The incidence of amyloidosis is twice as high in patients over 65 compared to those aged 35–54 [3,4,5,6]. Cardiac involvement is very common, which affects 50–70% of patients at diagnosis. [7,8,9]. The median overall survival (OS) of AL patients is approximately five years but more than 40% of patients die within the first year of diagnosis [10]. Prognosis for patients with advanced cardiac AL is poor, with a median OS of 4 months [11]. Depending on the clinical stage of AL, the median OS for stage I, II, IIIa, and IIIb of the European 2015 modification of Mayo 2004 classification is 130, 54, 24, and 4 months, respectively [12,13,14].

Early diagnosis of AL, the use of novel therapies, such as bortezomib and the anti-CD38 monoclonal antibodies, and, in a selected group of patients, high-dose chemotherapy followed by autologous stem cell transplantation (ASCT) improve the OS of patients with AL [15,16]. The worst prognosis is seen in a subgroup of patients with advanced cardiac involvement as defined by the European 2015 modification of Mayo 2004 classification, stages IIIa or IIIb, in which the median OS is 24 and 4 months, respectively [11,14]. Early diagnosis and appropriate treatment are essential to improve the OS of patients with severe cardiac AL.

Our multi-center retrospective study aimed at analyzing clinical characteristics, prognostic factors, and treatment outcomes in 67 unselected cardiac AL patients with the European 2015 modification of Mayo 2004 stage III disease [13,14]. 

## 2. Materials and Methods

### 2.1. Study Population

This multi-center, retrospective study was conducted in 13 centers from four European countries (Germany, Italy, Poland, and Spain), Chile, and the United States. Between July 2012 and August 2022, adult (≥18 years) patients with the European 2015 modification of the Mayo 2004 stage III cardiac AL were identified from the medical records at the participating study centers [13,14]. All centers had institutional review board approval. 

The diagnosis of AL amyloidosis and assessment of organ involvement was performed based on consensus criteria published in 2005 and modified in 2012 [17,18]. Amyloidosis AL was diagnosed based on clinical symptoms following applicable criteria and confirmed by tissue biopsy. The AL subtype was determined based on the presence of amyloid deposits in Congo-Red-positive fibril deposition and the green birefringence viewed under polarized light with concomitant clonality confirmed by the presence of a monoclonal protein in serum and/or urine, light-chain excess of serum-free light-chain (sFLC) test, and clonal plasma cells in the bone marrow.

Stage III of cardiac AL was defined according to the European 2015 modification of Mayo 2004 classification with stage IIIa defined as a serum concentration of cardiac troponin T (cTnT) ≥ 0.035 mcg/L or high-sensitivity cTnT (hs cTnT) ≥ 50 ng/L or cardiac troponin I (cTnI) ≥ 0.1 mcg/L and NT-proBNP 332–8499 ng/L; stage IIIb was defined as a NT-proBNP ≥ 8500 ng/L [13,14].

Patients with cardiac AL diagnosed with stage I, II, and other types of amyloidosis, monoclonal gammopathy of undetermined significance (MGUS), solitary plasmacytoma, multiple myeloma (MM), and other plasma cell dyscrasias were excluded from our analysis. The hematological and cardiac responses to treatment were determined according to published criteria [18,19]. The criteria used in MM were used to determine the cytogenetic risk. High-risk cytogenetic abnormalities were defined as t(4;14), t(14;16), t(14;20), del17p/p53 mutation, gain/amplification (1q), or ≥2 high risk cytogenetic abnormalities [20].

A hematologic complete response (CR) was defined as having no evidence of clonal disease by electrophoresis and immunofixation in serum or urine, with normal serum FLC levels and ratio. The difference between involved and uninvolved FLC level (dFLC) responses was evaluated, with a very good partial response (VGPR) defined as post-treatment dFLC level < 40 mg/L and a partial response (PR) by a 50% drop in dFLC serum level. The overall response rate (ORR) was defined as the achievement of at least a partial response (PR) or better. Obtaining less than a PR is referred to as no response (NR). Cardiac CR (carCR) was defined as nadir NT-proBNP ≤ 350 pg/mL, cardiac VGPR (carVGPR) as >60% reduction in NT-proBNP from baseline level, and cardiac PR (carPR) as 31–60% reduction in NT-proBNP from baseline level not meeting carCR. The cardiac overall response rate (carORR) was defined as the achievement of at least a partial response (PR) or better. A reduction in NT-proBNP concentration ≤ 30% compared to baseline values defines no cardiac response (carNR) [17,18,19].

Progression-free survival (PFS) was expressed in months and was defined as the time from diagnosis to disease progression, change in treatment, or death, whichever occurred first. Overall survival is described in months as the time from diagnosis until death or last follow-up. Early death was defined as death within 3 months of diagnosis. 

Univariate analysis of OS was performed for some essential variables, including age, Eastern Cooperative Oncology Group performance status (ECOG PS), New York Heart Association functional classification (NYHA FC), stage III (the European 2015 modification of Mayo 2004 classification), cytogenetic abnormalities at diagnosis, number of organs involved, and effectiveness of first-line treatment, including the use of ASCT as a part of the planned initial treatment sequence.

### 2.2. Statistical Analysis

Continuous and categorical variables are presented using descriptive statistics. The Kaplan–Meier (K-M) method analyzed survival and generated survival curves [21]. Time-to-event curves were plotted with the method of K-M, and comparisons among groups were made using the log-rank test. The Chi-square test was used to compare categorical variables. The Cox proportional-hazard regression method was used to fit univariate and multivariate survival models, reported as hazard ratios (HRs) with 95% confidence intervals (95% CIs). All reported *p*-values are two-sided and were considered significant if less than 0.05. 

Significant variables in the univariate analysis (*p* < 0.05) were included in the multivariate Cox regression analysis. The survival analyses did not include variables with >50% of missing data. Statistical analysis and graphics were obtained using the software PQStat version 1.8.6. and a package dedicated to survival analysis. 

## 3. Results

### 3.1. Patient Information

Sixty-seven patients with newly diagnosed modified Mayo (with the European 2012 modification of Mayo 2004 classification) stage III cardiac AL were included in the analysis. Patient characteristics and clinical features are listed in Table 1. The median follow-up was 10 months (range, 1–111). The median age at diagnosis of cardiac AL was 64 years (range, 41–83), with 31 (46.3%) ≥ 65 years of age and 11 (16.4%) ≥ 75 years. The majority of the patients were male (n = 36, 53.7%). Using the European 2015 modification of Mayo 2004 classification, 39 patients (58.2%) were diagnosed with stage IIIa and 28 (41.8%) were diagnosed with stage IIIb. Fifty-nine patients (88.1%) had additional organ(s) involvement. The median number of organs involved was two (range, 1–5). A total of 52 patients (77.6%) had sFLC type lambda and 15 patients (22.4%) had sFLC kappa. All patients were monitored using sFLC measurements. 

Cardiac biomarkers and echocardiography parameters are summarized in Table 2. The median value of N-terminal of pro-B-type natriuretic peptide (NT-proBNP) was 6832 ng/L (range, 655–70,000) for the, 54.5 ug/L (range, 0.05–397) for cTnT, 98.1 ng/L (range, 45–566) for high-sensitivity TnT (hs TnT), and 0.26 ug/L (range, 0.02–4.7) for cTnI. Echocardiography was performed in 64 patients (95.5%), with a median left ventricle ejection fraction (LVEF) of 58% (range, 30–75%). Cardiac MRI was performed in 31 patients (46.3%) and endomyocardial biopsy in 15 patients (22.7%). 

Baseline cytogenetics by fluorescent in situ hybridization (FISH) was available in 37 patients (55.2%), with high-risk cytogenetic abnormalities found in 12 patients (32.4%). Twelve patients (32.4%) had t(11;14).

### 3.2. Methods and Effectiveness of First-Line Treatment

All patients received at least one cycle of planned first-line chemotherapy. Treatment details are presented in Table 1. A total of 64 patients (95.5%) were treated with a bortezomib-based regimen; 28 patients (43.7%) received bortezomib in combination with dexamethasone (Vd), 25 patients (39.0%) received Vd in combination with cyclophosphamide (VCd), and 11 (17.3%) patients received Vd plus an immunomodulatory drug (IMiD), mostly thalidomide (VTd in 8 patients). Three patients (4.5%) received IMiD-based therapy, including one patient using thalidomide in combination with cyclophosphamide and dexamethasone (CTd) and two using lenalidomide combined with dexamethasone (Rd). Fourteen patients (20.9%) were treated with daratumumab-based regimens, including nine patients with VCd (Dara-VCd) and five with Vd (Dara-Vd). The median number of cycles of first-line therapy for the entire study group was 3.5 (range, 1–13). After induction therapy, 10 patients (14.9%) proceeded to ASCT based on the transplant eligibility criteria applicable at the individual centers participating in the study. Maintenance therapy after ASCT was used in four patients (one patient—lenalidomide, one—ixazomib, one—bortezomib plus daratumumab, and one—daratumumab). 

After first-line treatment, 52 patients (77.6%) had a hematological evaluation of treatment efficacy, 52 (77.6%) patients had a cardiac evaluation, and 50 (74.6%) had both hematologic and cardiac assessment of treatment efficacy. The hematologic ORR was 71.1%, and ≥VGPR was 46.1%; CR, VGPR, PR, and NR rates were 28.8%, 17.3%, 25.0%, and 28.9%, respectively. The carORR was 38.5%. The median time to achieve the best hematologic and cardiac responses were 6 months. Efficacy data for first-line therapy are summarized in Table 3.

**Figure 1:**
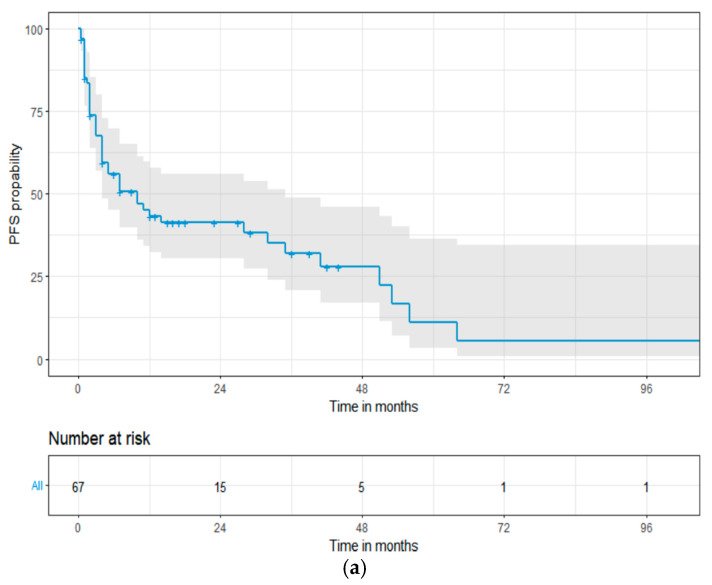

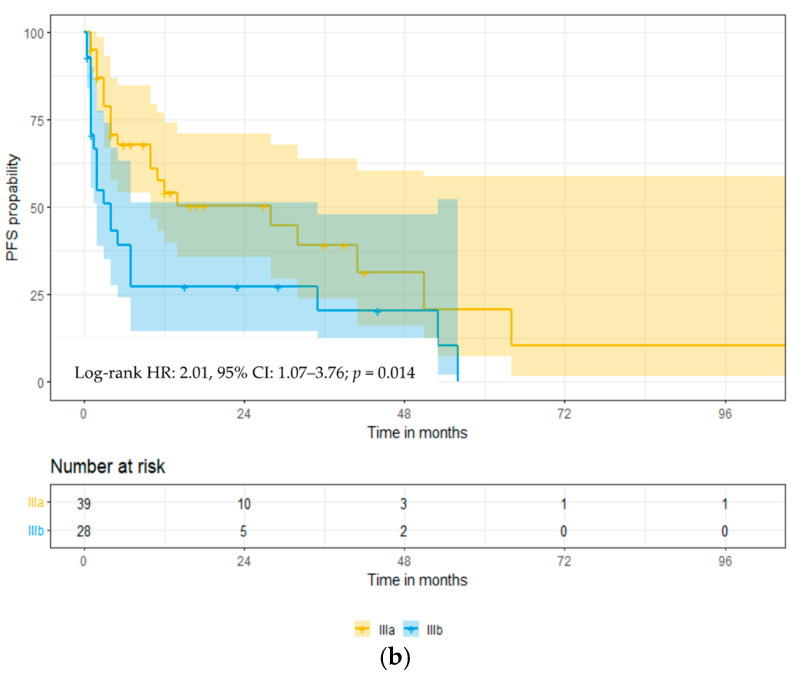
The Kaplan–Meier progression-free survival curves in 67 patients with the European 2012 modification of Mayo 2004 stage III cardiac light-chain amyloidosis (**a**), stage IIIa and IIIb (**b**). Abbreviations: HR: hazard ratio; PFS: progression-free survival.

The median PFS for all patients was 10 months (95% CI: 4–32, Figure 1a). Comparing patients in stage IIIa and IIIb, the median PFS was 28 vs. 4 months (log-rank HR: 2.01, 95% CI: 1.07–3.76; *p* = 0.013, Figure 1b), respectively. Additionally, significant prolongation of PFS was observed in patients who achieved a hematological VGPR or better and/or cardiac PR or better after first-line treatment; median PFS was 51 vs. 4 months (log-rank HR: 0.37; 95% CI: 1.19–0.73; *p* = 0.003) and 53 vs. 4 months (log-rank HR: 0.26; 95% CI: 0.13–0.52; *p* < 0.001), respectively. In the 14% of the patients who underwent an ASCT, a trend towards superior PFS was observed, with the median PFS being 51 vs. 7 months, respectively (log-rank HR: 1.78; 95% CI: 0.83–3.80; *p* = 0.196).

Eighteen patients (51.2%) received second-line therapy. Eight patients were treated with a Bortezomib-based therapy, seven patients received a daratumumab-based therapy, and three received a lenalidomide-based therapy. The ORR in the 15 evaluable patients was 80.0%; CR, VGPR, PR, and NR rates were 26.7%, 40.0%, 13.3%, and 20.0%, respectively. The median PFS was eight months (95% CI: 3–27). Due to the limited number of patients, the assessment of the effectiveness of second-line treatment was not statistically analyzed.

### 3.3. Overall Survival

**Figure 2:**
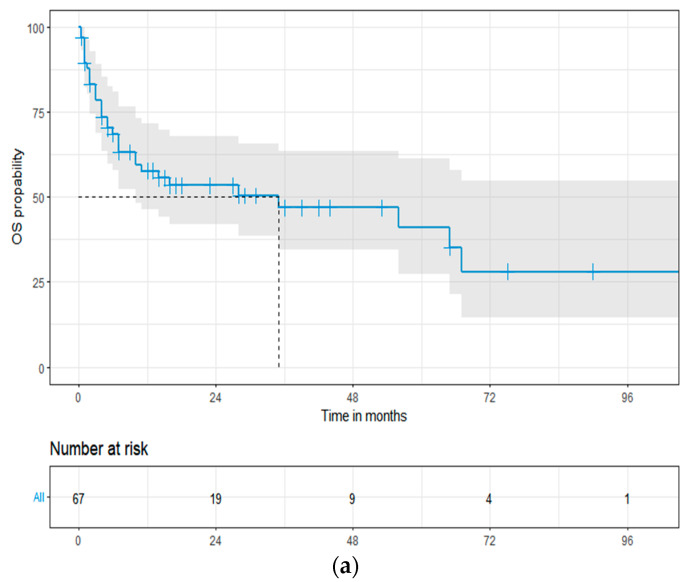

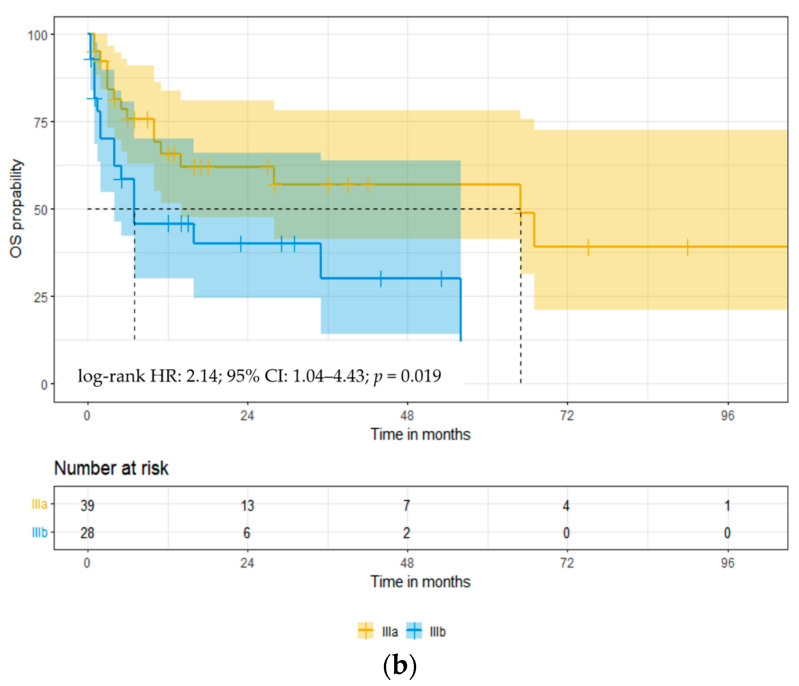
The Kaplan–Meier overall survival curves in 67 patients with the European 2012 modification of Mayo 2004 stage III cardiac light-chain amyloidosis (**a**), stage IIIa and IIIb (**b**). Abbreviations: HR: hazard ratio; OS: overall survival.

The median OS for the entire group was 35 months (95% CI: 7–67, Figure 2a). Significantly better OS was observed in patients with stage IIIa compared to stage IIIb; the median OS was 65 vs. 7 months (log-rank HR: 2.14; 95% CI: 1.04–4.43; *p* = 0.019, Figure 2b), respectively. At a median follow-up of 10 months, 33 patients (49.2%) had died, of which 13 (19.4%) died within three months of diagnosis. The most common cause of death was sudden cardiac death in 16 patients (48.5%), progressive disease (PD) in 10 patients (30.3%), infections in 3 patients (9.1%), and other causes in 4 patients (12.1%). Sudden cardiac death was the cause of death in 37.5% of patients with stage IIIa and in 61.5% of stage IIIb.

**Figure 3:**
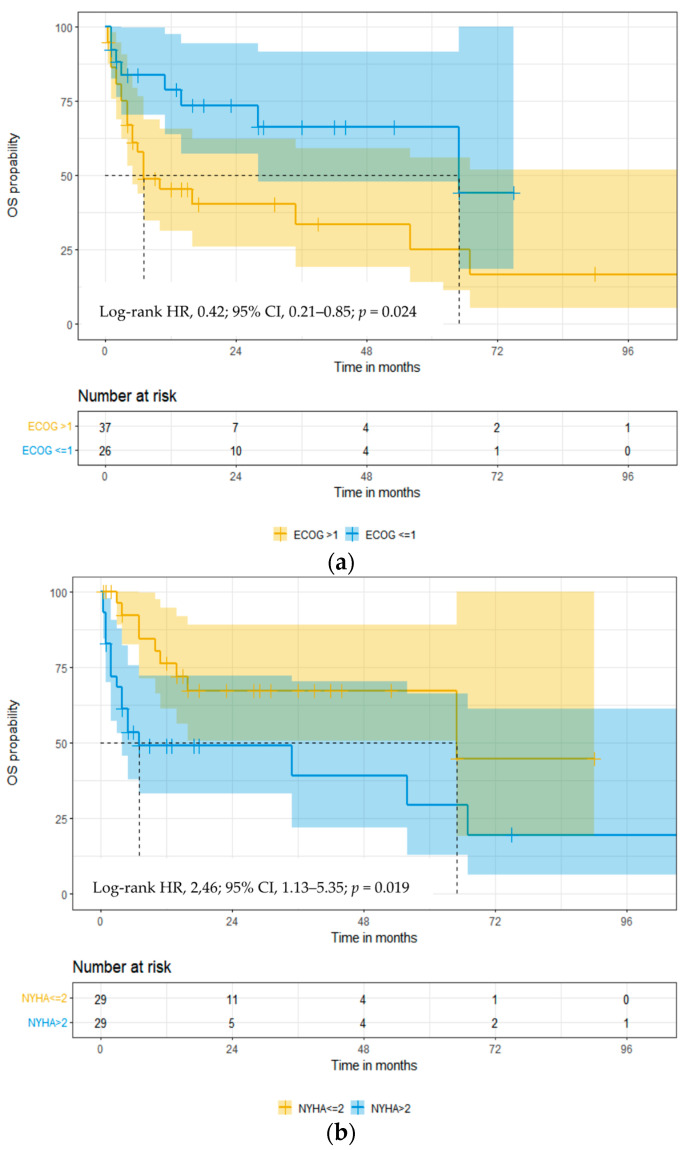

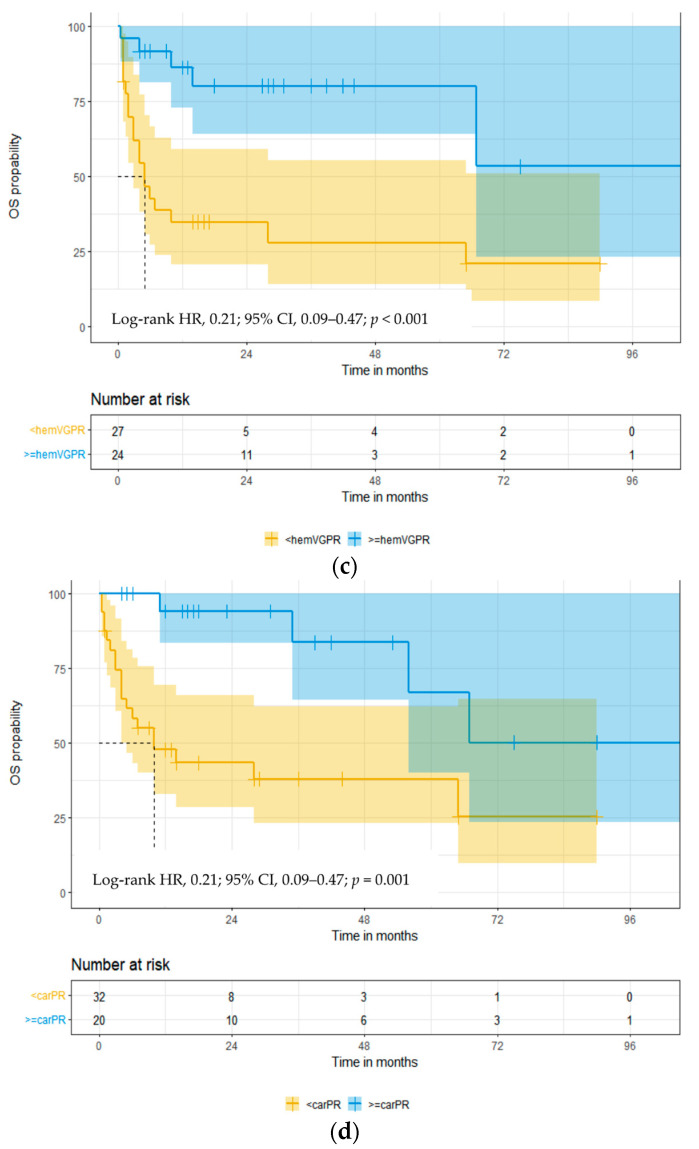

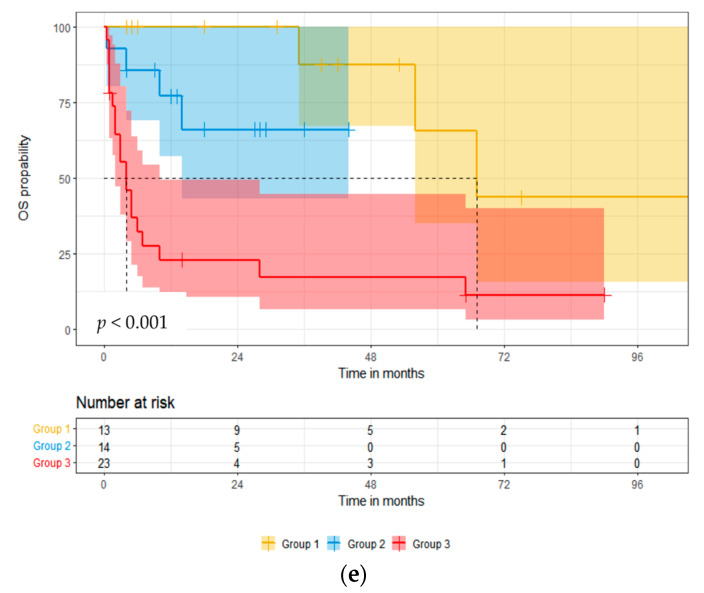
The Kaplan–Meier overall survival curves in 67 patients with the European 2012 modification of Mayo 2004 stage III cardiac light-chain amyloidosis by Eastern Cooperative Oncology Group Performance Status (ECOG) (**a**), NYHA FC (**b**), hematological response (**c**), cardiac response (**d**), and hematological + cardiac response vs. no response (**e**). Abbreviations: carPR: cardiac partial response; HR: hazard ratio; hemVGPR: hematological very good partial response; OS: overall survival; PR: partial response; VGPR: very good partial response. Group 1: ≥hematological VGPR and ≥cardiac PR; Group 2: ≥hematological VGPR and <cardiac PR; Group 3: <hematological VGPR and <cardiac PR.

When analyzing the impact of age on OS, a trend for improved OS was identified in patients < 65 years vs. ≥65 years of age; median OS was not reached (NR) vs. 14 months, respectively (log-rank HR: 0.57; 95% CI: 0.28–1.13; *p* = 0.095). A significant improvement in OS was evident with better performance status: the median OS of patients with ECOG PS ≤ 1 vs. >1 was 65 vs. 7 months (log-rank HR: 0.42; 95% CI: 0.21–0.85; *p* = 0.024, Figure 3a). Similarly, patients with better NYHA FC had a longer OS; median survival NYHA FC was <2 vs. >2, 65 vs. 7 months (log-rank HR: 2.46; 95% CI: 1.13–5.35; *p* = 0.019, Figure 3b), respectively. Patients with involvement of fewer organs showed a trend towards better OS in patients with ≤2 vs. >2 organ involvement: median OS was 36 vs. 6 months (log-rank HR: 2.05; 95% CI: 0.75–5.56; *p* = 0.059), respectively.

Regarding cytogenetic stratification, in the 37 evaluable patients, the OS of patients with the standard-risk cytogenetics (67.6%) was superior to high-risk cytogenetics (32.4%) with an OS of 56 vs. 10 months (log-rank HR: 2.51; 95% CI: 0.89–7.08; *p* = 0.051), respectively.

The first-line treatment was primarily bortezomib-based chemotherapy, including Vd (n = 24), VCd (n = 16), daratumumab + VCd (n = 9) regimens. The median OS in the three groups amounted to 35 vs. 10 vs. 16 months (*p* = 0.741). The OS differences in the three groups were not statistically significant, likely due to low numbers. In the ASCT group (n = 10), the median OS in the patients treated vs. untreated were NR vs. 14 months. The median OS of patients who received ASCT was significant (log-rank HR: 9.07; 95% CI: 3.98–20.64; *p* = 0.007), respectively. 

We analyzed OS according to the hematologic and cardiac response. The median OS in the group of patients who achieved hematologic response ≥ VGPR vs. <VGPR was NR vs. 5 months (log-rank HR: 0.21; 95% CI: 0.09–0.47; *p* < 0.001, Figure 3c). The median OS in the group of patients who achieved cardiac response ≥ PR vs. <PR was NR vs. 10 months (log-rank HR: 0.21; 95% CI: 0.09–0.47; *p* = 0.001, Figure 3d), respectively. We found the most prolonged OS in the group of patients who achieved both a hematological (≥VGPR) and cardiac response (≥PR); the OS medians were 67 vs. NR vs. 4 months (log-rank HR: 3.83; 95% CI: 1.43–10.21; *p* < 0.001, Figure 3e), respectively. 

**Figure 4:**
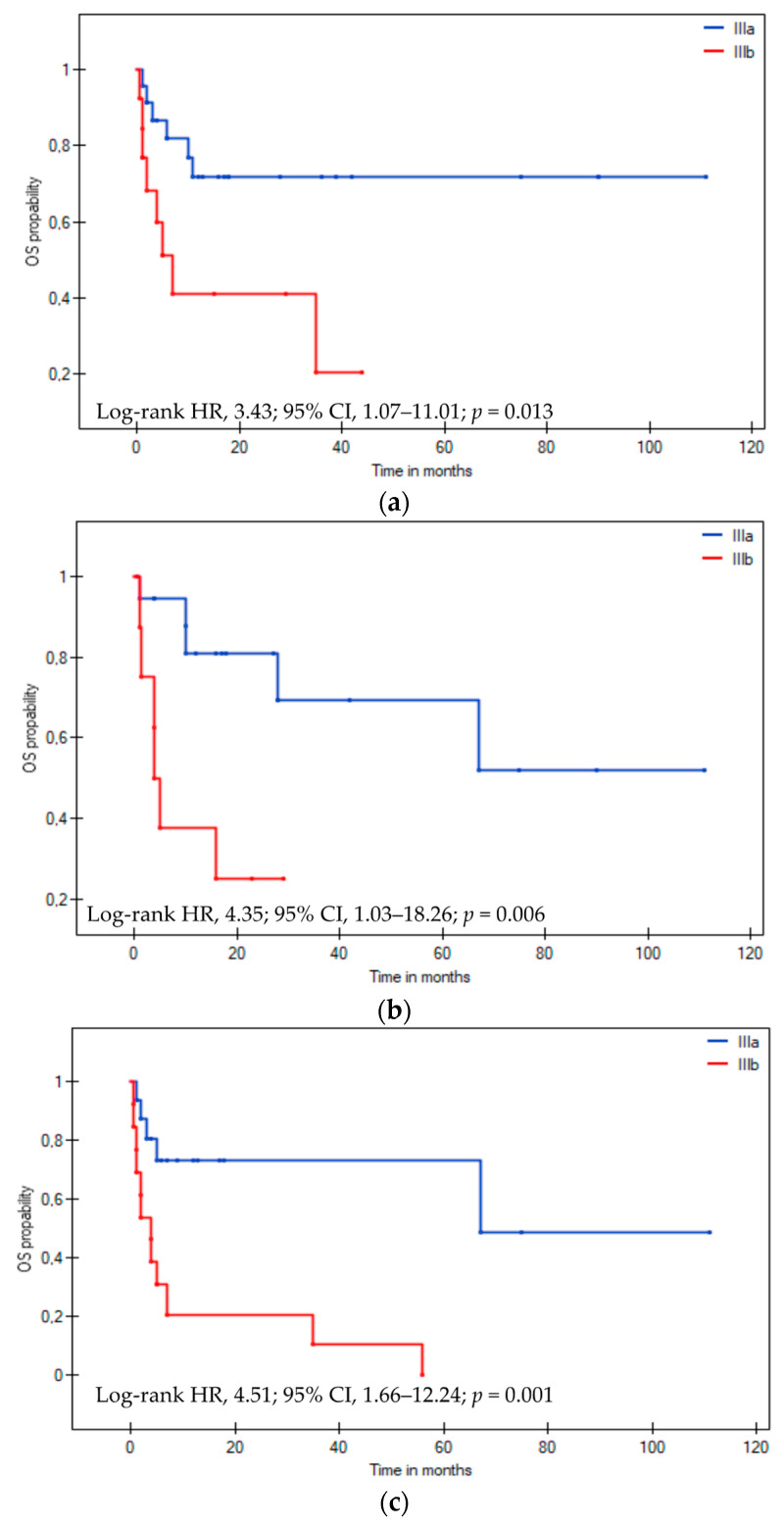

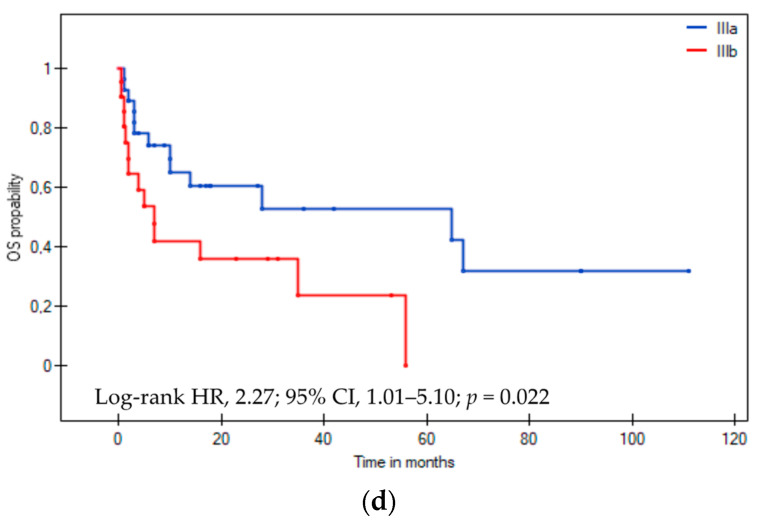
The Kaplan–Meier overall survival curves in patients with the European 2012 modification of Mayo 2004 stage IIIa (n = 39) and IIIb (n = 28) cardiac light-chain amyloidosis by age < 65 years (**a**), no comorbidities (**b**), NYHA FS > 2 (**c**), bortezomib-based treatment in first-line therapy (**d**). Abbreviations: HR: hazard ratio; NYHA FC: New York Heart Association Functional Classification; OS: overall survival.

Analyzing cardiac AL according to stages IIIa and IIIb, we observed that, in the group of patients younger than 65 years old, the median OS was NR vs. 7 months, respectively (log-rank HR: 3.43; 95% CI: 1.07–11.01; *p* = 0.013, Figure 4a), while, in the group of patients older than 65 years old, it was 16 vs. 14 months, respectively (log-rank HR: 1.10; 95% CI: 0.44–2.75; *p* = 0.833). In patients without comorbidities, the median OS in stage IIIa and IIIb was NR vs. 4 months, respectively (log-rank HR: 4.35; 95% CI: 1.03–18.26; *p* = 0.006, Figure 4b). We did not observe a similar relationship in the group of patients with comorbidities, where the median OS in stage IIIa and IIIb was 14 vs. 7 months, respectively (log-rank HR: 1.38; 95% CI: 0.59–3.21; *p* = 0.431). Considering ECOG PS, the median OS in stage IIIa and IIIb in patients with ECOG PS ≤ 1 was NR vs. 65 months, respectively (HR log-rank: 1.17; 95% CI: 0.22–6.24; *p* = 0.431), and, in patients with ECOG PS > 1, 10 vs. 7 months, respectively (HR log-rank: 1.78; 95% CI: 0.78–4.04; *p* = 0.148). Median OS in stage IIIa and IIIb depending on NYHA FC < 2 and NYHA FC ≥ 2 was 65 months vs. NR, respectively (log-rank HR: 0.77; 95% CI: 0.20–2.93; *p* = 0.697) and 67 vs. 4 months, respectively (log-rank HR: 4.51; 95% CI: 1.66–12.24; *p* = 0.001, Figure 4c). In the group of patients treated with bortezomib (Vd, VCd, and Vd + IMiD), the median OS in stage IIIa and IIIb was 65 vs. 7 months, respectively (log-rank HR: 2.27; 95% CI: 1.01–5.10; *p* = 0.022, Figure 4d).

Prognostic factors associated with OS in univariate and multivariate analyses are presented in Table 4. In a univariate analysis of ECOG PS, NYHA FC, Mayo stage, ASCT treatment in first-line therapy, hematologic and cardiac response, and gastrointestinal tract involvement were predictive. Multivariate analysis identified three independent factors: ECOG PS, NYHA FC, and hematologic and cardiac response.

## 4. Discussion

AL amyloidosis is a multisystemic disease that carries a high mortality risk for patients with an advanced degree of cardiac involvement.

Our retrospective study included 67 patients with the European 2012 modification of Mayo 2004 stage III cardiac AL. The prognosis for patients with stage III cardiac amyloidosis is very serious, especially among those with stage IIIb. Due to the small size of the study group, we performed a statistical analysis of combined subgroups, including stages IIIa and IIIb. We examined the clinical and laboratory factors and various treatment modalities and their effectiveness in influencing OS. Additionally, we analyzed progression-free survival (PFS) and overall survival (OS) for stages IIIa and IIIb, finding significantly better PFS and OS in stage IIIa. Significantly better OS was found in stage IIIa compared to IIIb among patients aged < 65 years, without comorbidities, with NYHA FC > 2, and treated with Bortezomib-based therapy.

In the Cox regression analysis, factors which were found to be associated with a favorable impact on OS were ECOG PS, NYHA FC, stage IIIa, utilization of ASCT, and the achievement of a hematological response ≥ VGPR and a cardiac response ≥ PR.

In one European retrospective study, higher proportions of patients (26.6% and 17.6% pts with stage IIIa and IIIb, respectively) were found to have an advanced degree of cardiac involvement. Per Palladini et al., the median OS for patients with stage IIIa and IIIb diagnosed before 2010 was 14.2 months vs. 5 months, respectively, while, in patients diagnosed with amyloidosis after 2010, the median OS was 30.7 vs. 4.5 months, respectively [9]. Prognostic markers such as NT-proBNP (≥8500 ng/L) and SBP (<100 mmHg) have been associated with a shorter OS [14]. Kristen et al. found that NYHA FC, glomerular filtration class, and treatment efficacy impact OS [22]. Other prognostic factors which have been evaluated include an advanced age, elevated cardiac troponin I (cTnI) levels, and LV systolic or diastolic dysfunction [23]. In contrast, our study did not identify any impact of laboratory factors on OS, except NT-proBNP ≥ 8500 μg/L.

In our study, the hematological ORR after first-line treatment was 71.4%, and the cardiac ORR was 38.5%. Treatment was heterogeneous but most patients received a bortezomib-based therapy. In our study, hematological responses were comparable to those found in both retrospective and prospective studies in which bortezomib-based treatment was used (68–71%) [24,25]. Venner et al. reported that the addition of cyclophosphamide to Vd resulted in a hematologic ORR of 94% and cardiac response of 29% [26]. The combination of melphalan with Vd has been found to produce a hematological response of 68–96% and a cardiac response of 32–60% [27,28]. Our analysis did not find a statistically significant difference in OS across groups that received chemotherapy regimens in the first-line setting (Vd vs. VCd vs. Dara-VCd). However, the addition of daratumumab to VCd has been shown to be superior to VCd in the larger phase 3 randomized Andromeda trial, where the reported hematologic and cardiac response rates were 92% and 41.5%, respectively, in the Dara-VCd arm compared to 77% and 22%, respectively, in the VCd arm [29]. In our study, daratumumab was used as first-line treatment in 14 patients. We did not find a significant effect on the prolongation of OS compared to patients who did not receive daratumumab, probably due to the small number of patients.

In both univariate and multivariate analyses, we found that the most significant impact on OS was achieved by the hematologic and cardiac response after first-line treatment.

Despite the implementation of therapy in all analyzed patients, 45% of patients died within the first three months of the diagnosis and the most common cause of death was related to cardiac events. This significantly differs from MM patients, where infections are the most common cause of early death [30].

The results of the treatment of cardiac AL are still unsatisfactory, especially for stage III. The use of new therapies, including monoclonal antibodies such as birtamimab (NEOD001) [31,32,33] or anselamimab (CAEL-101) [34,35,36,37,38], improves the prognosis in this group of patients but further randomized clinical trials are necessary.

Our study has several limitations. First, it is a retrospective study with a limited number of patients. Second, the chemotherapy protocols used for first-line therapy were heterogeneous. Due to the size of the study group, we did not analyze the impact of second-line therapy on survival. In addition, cytogenetic studies, and the use of ASCT were only available in a minority of patients. Additionally, the study did not determine the time from the onset of clinical symptoms to the initiation of AL treatment.

In conclusion, based on a series of patients with the European 2012 modification of Mayo 2004 stage III cardiac AL, we found that the prognosis in this group of patients is very poor and almost half of them die within three months of diagnosis.

## 5. Conclusions

Our study shows that factors associated with the most significant favorable impact on OS for patients with the European 2012 modification of Mayo 2004 stage III cardiac AL, identified by multivariate Cox analysis are ECOG PS ≤ 1, NYHA FC ≤ 2, and the achievement of a hematological response of ≥VGPR and a cardiac response of ≥PR after first-line treatment.

The development of newer treatment strategies as well as novel agents is necessary to improve the survival outcomes of patients with advanced heart failure as determined by the European 2012 modification of Mayo 2004 staging system, particularly those with stage III cardiac AL. Several trials are investigating the use of monoclonal antibodies such as birtamimab (AFFIRM-AL) and anselamimab (CAEL-101), which are directed against amyloid fibrils deposited in target organs.

## Figures and Tables

**Table 1:** Baseline clinical characteristics of patients with the European 2012 modification of Mayo 2004 stage III cardiac light-chain amyloidosis.

Parameters	All Patients (n = 67)	Stage IIIa (n = 39; 58.2%)	Stage IIIb (n = 28, 41.8%)
	n (%) or Median (Range)	n (%) or Median (Range)	n (%) or Median (Range)
Male, n (%)	36 (53.7)	24 (61.5)	12 (42.8)
Median age, years	64 (41–83)	63 (41–83)	66 (41–83)
Distribution			
<65 years	36 (53.7)	22 (56.4)	14 (50.0)
≥65 years	31 (46.3)	17 (43.6)	14 (50.0)
ECOG PS, n = 63			
≤1	26 (41.3)	19 (52.8)	7 (25.9)
>1	37 (58.7)	17 (47.2)	20 (74.1)
Comorbidities			
0/≥1	27 (40.3)/40 (59.7)	18 (46.1)/21 (53.9)	9 (32.1)/19 (67.9)
sFLC, n = 67			
lambda/kappa	52 (77.6)/15 (22.4)	32 (82.0)/7 (18.0)	20 (71.4)/8 (28.6)
Heavy chain, n = 67			
IgG/IgA/IgM	21 (31.3)/7 (10.4)/1 (1.5)	14 (35.9)/5 (12.8)/0 (0.0)	9 (32.1)/2 (7.1)/1 (3.6)
Other organ involvement			
Kidneys, n = 66	42 (63.6)	25 (64.1)	17 (63.0)
Liver, n = 59	10 (16.9)	5 (14.7)	5 (20.0)
Peripheral neuropathy, n = 60	10 (16.7)	6 (16.7)	4 (16.7)
Autonomic neuropathy, n = 59	13 (22.0)	6 (17.1)	7 (29.2)
Gastrointestinal tract, n = 56	9 (16.1)	5 (14.7)	4 (18.2)
Cytogenetics			
High-risk cytogenetics, n = 37	12 (32.4)	8 (38.1)	4 (25.0)
t(11;14)	12 (32.4)	7 (33.3)	5 (31.2)
First-line chemotherapy			
Bort-based	64 (95.5)	38 (97.4)	26 (92.8)
Vd/VCd/Vd + IMiD	28 (43.7)/25 (39.0)/11 (17.3)	15 (39.5)/15 (39.5)/8 (21.0)	13 (50.0)/10 (38.5)/3 (11.5)
IMiD-based (CTd, Rd)	3 (4.5)	1 (2.6)	2 (7.2)
Daratumumab	14 (20.9)	8 (20.5)	6 (21.4)
ASCT	10 (14.9)	9 (23.1)	1 (3.6)
Laboratory parameters			
CPBM > 10%, n = 67	20 (32.2)	13 (33.3)	7 (25.0)
Serum Hb (g/dL), n = 67	12.8 (7.7–17.5)	12.7 (10.6–16.2)	12.8 (7.7–17.5)
WBC count (×10^3^/μL), n = 67	8.4 (3.8–17.1)	8.2 (4.0–17.1)	8.7 (3.8–16.4)
PLT count (×10^3^/μL), n = 67	250.0 (114.0–624.0)	260.0 (117.0–620.0)	250.0 (114.0–624.0)
Serum albumin (mg/L), n = 67	3.2 (1.0–4.6)	3.3 (1.0–4.6)	3.2 (1.1–4.5)
Serum β2-microglobulin (mg/L), n = 49	4.0 (1.7–32.0)	3.0 (1.7–18.0)	5.5 (2.6–32.0)
sFLC lambda (mg/dL), n = 66	56.9 (0.7–7411.8)	54.8 (0.7–1493.0)	52.0 (0.9–7411.8)
sFLC kappa (mg/dL), n = 64	15.8 (0.2–3730.0)	13.6 (0.5–588.0)	16.0 (0.2–3730.0)
Serum LDH (IU/L), n = 49	267.0 (161.0–1021.0)	250.0 (170.0–758.0)	277.0 (161.0–1021.0)
Baseline echocardiography	64 (95.5)	39 (100.0)	25 (89.3)
Baseline cardiac magnetic resonance	31 (46.3)	22 (56.4)	9 (32.1)
Baseline endomyocardial biopsy	15 (22.7)	7 (17.9)	8 (28.6)

Abbreviations: CPBM: clonal plasma cells in bone marrow; CTd: cyclophosphamide, thalidomide, dexamethasone; ECOG PS: Eastern Cooperative Oncology Group Performance Status; Hb: hemoglobin concentration; Ig: immunoglobuline; IMiD: immunomodulatory drug; LDH: lactate dehydrogenase; PLT: platelets; sFLC: serum free light chain; WBC: white blood cells; VCd: bortezomib, cyclophosphamide, dexamethasone; Vd: bortezomib, dexamethasone.

**Table 2:** Cardiac parameters in patients with the European 2012 modification of Mayo 2004 stage III cardiac light-chain amyloidosis.

Parameters	All Patients (n = 67)	Stage IIIa (n = 39; 58.2%)	Stage IIIb (n = 28; 41.8%)
	n (%) or Median (Range)	n (%) or Median (Range)	n (%) or Median (Range)
NYHA FC, grade, n = 58			
1	3 (5.2)	2 (6.3)	1 (3.8)
2	26 (44.8)	14 (43.7)	12 (46.2)
3	24 (41.4)	15 (46.9)	9 (34.6)
4	5 (8.6)	1 (3.1)	4 (15.4)
SBP (mmHg), n = 43	105 (32–152)	105 (32–135)	110 (70–152)
<100 mmHg	16 (37.2)	10 (37.0)	6 (37.5)
LVEF (%), n = 60			
<50%	14 (20.9)	9 (25.0)	5 (20.8)
≥50%	46 (68.7)	27 (75.0)	19 (79.2)
Cardiac Troponin T (μg/L), n = 24	54.5 (0.05–397.0)	58.0 (0.07–242.0)	97.8 (0.05–397.0)
≥0.025 μg/L	24 (100.0)	15 (100.0)	9 (100.0)
High sensitivity Troponin T (ng/L), n = 22	98.1 (45.0–566.0)	68.5 (45.0–298.0)	220.4 (57.0–566.0)
≥40 ng/L	22 (100.0)	12 (100.0)	10 (100.0)
Cardiac Troponin I (μg/L), n = 19	0.26 (0.02–4.7)	0.23 (0.02–4.7)	0.35 (0.04–0.36)
≥0.1 μg/L	15 (78.9)	9 (75.0)	6 (85.7)
NT-proBNP (ng/L), n = 67	6832.0 (655.0–70,000.0)	4376.0 (655.0–8081.0)	16,011.0 (8735.0–70,000.0)
≥8500 μg/L	28 (41.8)	0 (0.0)	28 (100.0)

Abbreviations: LVEF: left ventricular ejection fraction; NT-proBNP: N-terminal fragment of B-type natriuretic peptide; NYHA FC: New York Heart Association Functional Class; SBP: systolic blood pressure.

**Table 3:** Hematological and cardiac responses after first-line therapy.

Response	All Patients, n (%)	Stage IIIa, n (%)	Stage IIIb, n (%)
Hematological responses, n = 52			
Overall response rate	37 (71.1)	24 (75.0)	13 (65.0)
Complete response	15 (28.8)	12 (37.6)	3 (15.0)
Very good partial response	9 (17.3)	6 (18.7)	3 (15.0)
Partial response	13 (25.0)	6 (18.7)	7 (35.0)
No response	15 (28.9)	8 (25.0)	7 (35.0)
Cardiac responses, n = 52			
Overall response rate	20 (38.5)	13 (40.6)	7 (35.0)
Complete response	3 (5.8)	3 (9.4)	0 (0.0)
Very good partial response	2 (3.8)	0 (0.0)	2 (10.0)
Partial response	15 (28.8)	10 (31.2)	5 (25.0)
No response	32 (61.5)	19 (59.4)	13 (65.0)
Hematological and cardiac response, n = 50			
≥hematological VGPR +≥cardiac PR	13 (26.0)	10 (33.3)	3 (15.0)

Abbreviations: PR: partial response; VGPR: very good partial response.

**Table 4:** Univariate and multivariate analyses for overall survival in patients with the European 2012 modification of Mayo 2004 stage III cardiac light-chain amyloidosis.

Parameters	Univariate Analysis		Multivariate Analysis	
	HR (95% CI)	*p* Value	HR (95% CI)	*p* Value
Age ≥ 65 years	1.76 (0.88–3.53)	0.104		
Male	1.06 (0.53–2.11	0.867		
Comorbidities ≥ 1	1.39 (0.81–2.38)	0.226		
ECOG PS > 1	0.41 (0.18–0.92)	0.032	0.23 (0.06–0.84)	0.026
NYHA FC > 2	2.54 (1.12–5.79)	0.025	4.33 (1.09–17.21)	0.037
Stage IIIb at diagnosis	2.25 (1.10–4.60)	0.025	1.65 (0.56–4.84)	0.36
Clonal plasma cells in bone marrow ≥ 10%	0.88 (0.41–1.86)	0.739		
High-risk cytogenetic abnormalities	2.62 (0.96–7.17)	0.061		
ASCT treatment	3.05 (1.12–8.25)	0.028	0.35 (0.04–2.79)	0.33
Hematologic response ≥ VGPR	0.21 (0.08–0.56)	0.002	0.26 (0.08–0.83)	0.023
Cardiac response ≥ PR	0.19 (0.06–0.58)	0.003	0.06 (0.01–0.34)	0.001
Hematologic response ≥ VGPR + Cardiac response ≥ PR	3.89 (1.79–8.41)	<0.001	4.30 (1.97–9.38)	<0.001
Renal involvement	0.97 (0.48–1.99)	0.945		
Liver involvement	1.81 (0.68–4.82)	0.236		
GI tract involvement	2.44 (1.03–5.80)	0.042	not included	
PN involvement	1.45 (0.62–3.40)	0.393		
Organs involved > 2 at diagnosis	0.53 (0.18–1.52)	0.238		
Hb < 12 g/dL	1.38 (0.67–2.83)	0.383		
SBP < 100 mmHg	0.52 (0.18–1.45)	0.209		
Serum albumin < 3.5 g/dL	1.46 (0.65–3.29)	0.352		
Serum β2-microglobulin ≥ 5.5 mg/L	1.46 (0.66–3.25)	0.346		
LDH > ULN	2.47 (0.81–7.49)	0.108		

Abbreviations: ASCT: autologous stem cell transplantation; ECOG PS: Eastern Cooperative Oncology Group Performance Status; GI: gastrointestinal; Hb: hemoglobin concentration; HR: hazard ratio; LDH: lactate dehydrogenase; NYHA FC: New York Heart Association Functional Classification; PN: peripheral neuropathy; PR: partial response; SBP: systolic blood pressure; ULN: upper limit of normal value; VGPR: very good partial response.

## Data Availability

Data are contained within the manuscript. Raw data are available upon reasonable request from the corresponding author. The data are not publicly available due to ethical restrictions.
